# Psychological, Physical and Behavioral Health of Adults, 3 Years After Exposure to a Train Derailment

**DOI:** 10.1177/00469580221125765

**Published:** 2022-09-30

**Authors:** Danielle Maltais, Mélissa Généreux, Mathieu Roy, Geneviève Fortin, Eve Pouliot, Christiane Bergeron-Leclerc, Jacques Cherblanc, Oscar Labra, Lise Lachance, Linda Paquette

**Affiliations:** 1 Université du Québec à Chicoutimi, Saguenay, QC, Canada; 2 Université de Sherbrooke, Sherbrooke, QC, Canada; 3 Université du Québec en Abitibi-Témiscamingue, Campus Rouyn-Noranda, Rouyn-Noranda, QC, Canada

**Keywords:** technological disaster, train derailment, post-disaster mental health, post-traumatic stress disorder, post-traumatic growth, drinking patterns

## Abstract

In July 2013, a train derailment profoundly disrupted the tranquility of the population of Lac-Mégantic for months and even years. In 2016, we conducted a representative population-based survey among 387 people from Lac-Mégantic and 413 from other municipalities with the aim to document psychological and physical health of adults exposed to the disaster. This article examines differences between 3 groups of respondents: those who were highly, moderately or not exposed to the train accident. Khi Square analyses, odds ratios and logistic regressions were used to examine differences between the 3 groups of respondents (high, moderate and no exposure). Results show that the level of exposure to this technological disaster is strongly associated with psychological suffering, post-traumatic growth, physical heath, drinking patterns, and use of prescribed and non-prescribed drugs. We can explain these results by the nature and cause of the event as well as its consequences.

**What do we already know about this topic?**
We know very little about the consequences of railway accidents that result in several deaths in a community with a small population. But we do know that when human negligence caused an accident, the consequences for the overall health of individuals can be numerous and long-lasting.
**How does your research contribute to the field?**
This research examines the effects of a severe train derailment on the mental health of individuals, depending on their level of exposure. The study also presents the sociodemographic and contextual factors associated with the presence of anxiety, depression, psychological distress, post-traumatic growth, and post-traumatic stress disorder.
**What are your research’s implications toward theory, practice, or policy?**
This study demonstrates the importance of conducting studies, several years after a technological disaster, integrating several variables that allow us to draw up a portrait of the health of the population and to identify the factors that public authorities must take into account to promote the recovery of individuals.

On the night of July 6, 2013, a train comprised of 63 tank cars carrying crude oil derailed in the downtown area of Lac-Mégantic^i^ (QC, Canada).^1^ The derailment triggered explosions, burning a large portion of the city center, killing 47 people and destroying 66 homes and 44 businesses. This event also forced the evacuation of over 2000 people, of a population of 6000 inhabitants. The entire population experienced the impact of this technological disaster, caused by a lack of railway tracks maintenance and human neglect, among others. The entire population experienced the impact of this technological disaster, caused by a lack of railway tracks maintenance and human neglect, among others. More than 3 years after the disaster, the government has not decided on building or not a bypass road. The community only received a few financial resources to rebuild the downtown that was largely ruined by the explosions.^2^ In order to document the medium-term repercussions of this disaster, a telephone survey was conducted in 2016 with 800 randomly selected respondents. Among these respondents, 387 adults lived in Lac-Mégantic and 413 in the other municipalities of the Regional County Municipality of Granit. During these interviews, the participants were asked to complete a questionnaire about their health status 3 years after the train derailment. This article reviews the physical, psychological and behavioral health status of adults who were exposed to this event.

## Background

In cross-sectional studies with a control group, the presence of post-traumatic stress disorder (PTSD) is strongly associated with the severity of the disaster and the level of exposure.^3,4^ The impact of disasters, whether natural or technological, is therefore proportional to the individual’s proximity to the location where the event occurred.^5^ In Toulouse (France), residents affected by fires following accidental explosions in a factory in 2000 were, 18 and 24 months after this event, 2 to 3 times more likely to show problems of depression, anxiety and sleep disorders than respondents in a control group.^6^ Furthermore, nearly half the workers exposed to these explosions were still experiencing psychological distress 2 years after the event.^7^ Despair and sleep and concentration difficulties are also significantly associated with geographic proximity to the disaster.^8^ Being evacuated because of severe fires is also related to post-traumatic stress disorder.^9^

Research has shown that technological disasters (eg, transportation accidents, fires, explosions, chemical and radioactive releases) are associated with the presence of post-traumatic stress, depression, anxiety and somatization.^4,10,11^ In addition, victims of this type of disaster may display several unhealthy behaviors usually associated with post-traumatic stress disorder, such as alcohol, substance and medication abuse.^12-15^ Norris et al^16^ indicate that the most frequent complaints about physical health are acute suffering (eg, pain, gastrointestinal problems, distress) and deterioration of overall health. Other physical health consequences resulting from disasters are abdominal pain, vomiting, nausea, paralysis, visual disturbances, fainting, headaches, fatigue, dizziness and concentration problems, as well as joint or muscle pains.^16,17^ Moreover, physiological reactivity, hypervigilance and outbursts associated with post-traumatic stress disorder may be related to cardiovascular changes such as hypertension, increased heart rate and arrhythmia.^18^ Women, seniors^19^ and those severely exposed to a disaster^16^ are more likely to be affected by post-disaster physical health problems.

The presence of psychological health problems after a disaster is associated with: (a) individual vulnerability, (b) exposure to the disaster, and (c) secondary stressors.^20-22^ The impact of a disaster does not stop once it is under control. In such a context, it is reasonable to anticipate, over 3 years after a train crash resulting in numerous deaths and widespread damage to individual and collective property, persistent differences between people exposed to the disaster and those not exposed. After a disaster, however, although some people report that their capacities are reduced compared to before the disaster, others say that the event strengthened them.^23^ Indeed, it has been shown that a disaster can also lead to post-traumatic growth for those exposed to it.^24-26^

Until now, limited research has been done on the relationship between traumatic exposure and alcohol use. In the scientific literature, results are inconsistent.^27^ Secondary stress factors, such as relocation, economic losses and changes in daily-life patterns, could be factors related to heavy drinking following a tragedy.^27^ In this regard, Boscarino et al^28^ showed that greater exposure to the World Trade Center disaster was significantly associated with alcohol abuse, which is consistent with findings from other studies on this event.^29,30^ After depression, the most commonly reported disorders associated with post-traumatic stress disorder are substance abuse disorders and particularly alcohol.^12^ The self-medication hypothesis seems most plausible to explain this phenomenon.^31,32^

As for medication, 6 months after an earthquake in Italy, researchers reported an increase of 129% in prescriptions for antipsychotics and 37% for antidepressants.^33^ Similarly, a short-term increase in the use of psychotropic drugs has been observed following disasters or terrorist acts.^34,35^

The present study aimed to (1) document the psychological and physical health of people who experienced the train derailment according to their exposure level and (2) identify predictive factors of the levels of post-traumatic stress disorder, psychological distress, depressive episode, post-traumatic growth, and an increase in alcohol or non-prescription drugs consumption. We hypothesize that respondents’ psychological and physical health status and lifestyle habits will vary according to the exposure level to the train derailment.

## Method

### Nature of the Study

This study is based on a one-time telephone survey consisting of close-ended questions. It is a cross-sectional population study; the data were analyzed using quantitative methods.

### Procedure

In fall 2016, a professional polling firm randomly recruited 387 adults living in Lac-Mégantic and 413 in the other municipalities of the Regional County Municipality (RCM) of Granit. A convenience sample of residential phone numbers was randomly created (Random Digit Dialing), in which residential and cell phone numbers were included and commercial numbers excluded. Before data collection, numbers were validated to exclude out-of-service numbers. From this list, we selected participants as follows: (1) random selection of households, (2) admissibility confirmation of households (living in Lac-Mégantic or other cities in the Granit MRC, at least one person >18 years old in the household), and (3) random selection of a person aged >18 years old in each household. We asked selected individuals to complete a 30-minutes questionnaire by phone. If the respondent was not available, we found a more appropriate moment to conduct the interview. The response rates were 47.8% for residents living in Lac-Mégantic and 50.1% for the other municipalities in the RCM of Granit.

The purpose of the questionnaire was to assess the intensity of the respondents’ exposure to the train derailment and to provide a portrait of their physical and psychological health and their use of alcohol, anti-anxiety drugs, antidepressants and non-prescription drugs 2.5 years after the tragedy. To minimize the non-response bias, the data on the consequences of the train derailment were weighted by assigning to each respondent a weight corresponding to the number of individuals they represented in the population.

### Participants

A total of 282 men and 518 women participated in this study. The regressions used for data analyses were requiring a sample size between 56 and 774, which confirms that the size of our sample is sufficient. G-power software was used for these calculations.

The majority of the respondents were married (63.8%), while 49.1% were employed either full-time or part-time, and a third were 65 years of age or older (32.3%) and had modest incomes (33.8%). Based on the participants’ responses regarding the losses experienced during the train derailment, the respondents were classified in 3 categories: highly exposed (24.8%), moderately exposed (53.2%) or not exposed to the tragedy (22.0%). Highly exposed respondents experienced human losses (fears for their life or that of a loved one, loss of a loved one or injury) and material damage (relocation or damage to their home) and viewed the train derailment as a stressful event that had negative impacts on their lives (subjective perception of the tragedy). In contrast, moderately exposed respondents experienced 2 of the 3 types of losses mentioned above (human, material and subjective), while those not exposed did not experience any of the 3 types of losses.

Table 1 shows that those who are (highly or moderately) exposed to the train derailment mainly come from the municipality of Lac-Mégantic (*P* < .001). Highly exposed respondents had the lowest percentage of people aged 65 or older (*P* < .05). There is, however, no significant difference in the percentages of respondents aged 18 to 49 and 50 to 64. Significant differences exist between respondents with respect to their marital status and the presence or absence of children aged 18 and under at home. For example, there are significantly more single people among non-exposed respondents than those moderately exposed to the train derailment (*P* < .05). In addition, almost twice as many highly exposed people (26.3%) compared to non-exposed people (14.8%) reported having children under 18 years of age (*P* < .05). However, there is no significant difference in the financial status and education of participants.

**Table 1.** Socio-Demographic Characteristics of Respondents by Level of Exposure (%).

Variables	Level of exposure			
	Highly exposed (n = 198)	Moderately exposed (n = 426)	Unexposed (n = 176)	Significance level (Khi-square) χ^2^
Place of residence				0.000***
Lac-Mégantic	82.8	45.1	17.6	
Elsewhere in MRC du Granit	17.2	54.9	82.4	
Gender				0.29
Man	30.8	36.2	38.1	
Woman	69.2	63.8	61.9	
Age				0.019*
18-49 years old	35.1	28.1	25.3	
50-64 years old	40.2	36.8	33.9	
65 years old and +	24.7	35.1	40.8	
Lives alone				0.06
Yes	31.8	23.1	27.8	
No	68.2	76.9	72.2	
Marital status				0.012*
Married/Free union	60.6	67.8	58	
Single	15.7	13.1	23.9	
Separated/Divorced	15.2	11	8	
Widowed	8.6	8	10.2	
Presence of children under 18 years of age				0.022*
Yes	26.3	22.8	14.8	
No	73.7	77.2	85.2	
Source of income				0.212
Full-time worker	43.4	38.7	34.1	
Part-time worker	12.1	9.7	9.7	
Retired	31.8	41.5	44.9	
Others	12.6	10.1	11.4	
Highest education level completed				0.288
Less than Secondary V	15.7	16.4	22.2	
Diploma of vocational studies	10.6	9.6	10.8	
Secondary	33.8	27.9	28.4	
College	20.7	22.1	23.3	
University	16.7	22.3	13.1	
Others	2.5	1.6	2.3	
Annual household income				
Less than $30 000	35.2	28.7	37.6	0.079
Between $30 000 and $79 999	50	53.2	52.1	
Over $80 000	14.8	18.1	10.3	

* *P* < .05. ***P < .001.

### Measures

We developed a questionnaire based on different tests with good psychometric properties. We validated all the scales and individual questions in previous studies with more than 1600 adults, 1 and 2 years after the derailment.^4,5,10,11,36-38^ The questionnaire included 62 dichotomous or multiple-choice questions. Questions aimed to collect information about the participants’ socio-demographic characteristics and level of exposure to the train derailment.

We assessed the presence of depressive episodes by 2 questions that asked if the respondents had been sad, melancholic, or depressed and experienced a loss of interest in the things they used to like for a consecutive period of 2 weeks or more in the past 12 months. The respondents also had to answer 2 questions asking them (1) whether they had a mood disorder, such as depression, bipolar disorder, mania, or dysthymia and (2) whether they had an anxiety disorder, such as a phobia, obsessive-compulsive disorder, or panic disorder. We previously used these questions in 2 population surveys conducted in 2014 and 2015 by Eastern Townships Public Health Department.^39^

The respondents’ substance use patterns were recorded based on questions about whether they used tranquilizers, sedatives or antidepressants prescribed by a doctor and the consumption frequency of 5 or more glasses of alcohol during a single occasion in the last 12 months. According to the *Association pour la santé publique du Québec*,^40^ this type of alcohol consumption is called “abusive consumption.” Respondents also had to estimate whether their consumption frequency had remained stable, decreased or increased over the last 3 years since the train tragedy.

The original version of Horowitz’s Impact of Event Scale^41^ was used to measure the presence or absence of post-traumatic stress disorder. The score of this instrument ranges from 0 to 75 points. A score greater than 25 indicates a moderate (26-43) or high level (44 or more) of post-traumatic stress disorder. In this study, the Alpha coefficient is .92 for the overall score.

The 6-item Kessler et al Psychological Distress Scale (K6) was used to assess the psychological distress of the respondents.^42,43^ Each of the 6 items is evaluated on a four-point scale, for a total score ranging from 0 to 24. People who score 7 or more are considered to have a clinical level of psychological distress.^44^ In this study, the Alpha coefficient is .84 for the overall score.

The Post-Traumatic Growth Inventory (PTGI) of Tedeschi and Calhoun and Calhoun^45^ was selected as a measure of post-traumatic growth. This test contains 21 questions aimed at defining the positive impacts of exposure to traumatic events in 5 areas: (a) relationships with others (7 items; Cronbach’s alpha *α* = .91); (b) new possibilities (5 items; *α* = .88); (c) personal strengths (4 items; *α* = .86); (d) appreciation of life (3 items; *α* = .83); and (e) spiritual changes (2 items; *α* = .66). This tool offers 6 answer choices ranging from 0 (“I never experienced this change”) to 5 (“I experienced this change very strongly”). A score greater or equal to 57 (out of a possible 105 points) indicates the presence of posttraumatic growth.^46^ The PTGI remains one of the most used tools among the 7 existing measuring instruments for measuring the presence or absence of posttraumatic growth.^47^ In this study, the Alpha coefficient is .96 for the overall score.

### Data Analysis

Statistical analyses were conducted using SPSS software (IBM SPSS Statistics for Windows, Version 26.0. Armonk, NY: IBM Corp). Descriptive analysis was conducted using chi-square tests. The groups were established based on their disaster exposure level (no exposure, moderate exposure and high exposure). When significant differences were identified between the groups, post-hoc comparative tests were conducted using the Bonferroni correction. For the physical and psychological health variables and for alcohol and medication consumption, odds ratios were computed to compare the risks associated with exposure to the train derailment, regardless of the level of exposure. A 95% confidence interval was used to account for the number of variables studied.

Hierarchical stepwise logistic regression analysis was conducted to account for the common variance between exposure and socio-demographic variables, social support variables and mental health outcomes. In the logistic regressions conducted, variables were introduced into models in 3 blocks: socio-demographic characteristics, social support and exposure variables. Of the 6 dependent variables used to assess the psychological state of the participants and their substance use patterns, 3 had continuous scores. Based on the flattening and asymmetry tests, only the post-traumatic stress variable met the normality criterion. In order to compare the results obtained for the different dependent variables, they were dichotomized from the valid split points for each instrument. The contribution of each block to the total percentage of explanation was reported for each model using the Nagelkerke squared correlation coefficient (*R*^2^).

## Results

### Perceived Physical and Psychological Health of Respondents by the 3 Level of Exposure

Three years after the tragedy, the majority of respondents, regardless of their level of exposure to train derailment, rate their physical health as excellent or very good (see Table 2). However, more highly and moderately exposed individuals than non-exposed respondents consider their health “fair” or “poor” (*P* < .05). Moreover, significantly more people who were highly exposed to the tragedy (compared to the other 2 groups of respondents) feel that their health has deteriorated or improved over the past 3 years (*P* < .01). The majority of highly exposed respondents (68.0%) also showed signs of post-traumatic stress disorder during the data collection. This percentage is significantly higher (*P* < .001) than that found in moderately exposed individuals (39.2%) and those not exposed to the tragedy (8.5%). Highly exposed individuals are also significantly more likely than the other 2 groups of respondents to have experienced anxiety (*P* < .001) or mood disorders (*P* < .001) in the year before the survey. In addition, moderately exposed individuals are more likely than unexposed individuals to experience the same psychological health problems (*P* < .001). Those who were highly or moderately exposed to the train derailment also had more depressive episodes and psychological distress in the last 12 months than those who were not exposed (*P* < .001). This study also found that those who were highly exposed were significantly more likely to have consulted a psychologist than the other 2 groups of respondents while consulting a social worker varied according to the level of exposure to the tragedy (*P* < .001).

**Table 2.** Perceived Physical and Psychological Health of Respondents by Level of Exposure (%).

Variables	Exposure level				Bonferroni correction
	Highly exposed(n = 198)	Moderately exposed(n = 426)	Unexposed(n = 176)	Significance level (Khi-square) χ^2^	
*State of physical health*					
Perceived health status				0.016*	HE, ME < U
Excellent to very good	80.8	82.9	90.9		
Fair to poor	19.2	17.1	9.1		
Changes in health level					HE, ME < U
Has improved	15.3	7.6	5.7	0.001***	
Remained stable	46.8	65.5	78.9		
Has deteriorated	37.9	26.9	15.4		
Consulting a professional					
Family doctor				0.225	
Yes	82.3	80.8	75.6		
No	17.7	19.2	24.4		
Medical specialist				0.003***	ME < U
Yes	40.1	45	30.1		
No	59.9	55	69.9		
*State of psychological health*					
State of post-traumatic stress disorder				0.000***	HE, ME < U
Yes (26 and over)	68	39.2	8.5		
No (25 and less)	32	60.8	91.5		
Presence of a mood disorder					HE, ME < U
Yes	17.7	9.5	2.8	0.000***	
No	82.3	90.5	97.2		
Presence of anxiety disorder				0.000***	HE, ME < U
Yes	27.1	13.3	2.8		
No	72.9	86.7	97.2		
Psychological distress				0.000***	HE, ME > U
Yes	37.4	28.3	10.2		
No	62.6	71.7	89.8		
Depressive episode				0.000***	HE, ME > U
Yes	40.4	31.4	14.8		
No	59.6	68.6	85.2		
*Consulting a professional*					
Psychologist				0.000***	HE, ME < U
Yes	15.3	8.1	4		
No	84.7	91.9	96		
Social worker				0.000***	HE, ME < U
Yes	18.7	10	4		
No	81.3	90	96		

* *P* < .05. ***P < .001.

Highly exposed individuals (15.3%) were significantly more likely (*P* < .01) than those moderately exposed (7.4%) or unexposed (4.0%) to estimate that their alcohol consumption has increased over the past 3 years. Highly exposed individuals are also significantly more likely than the other 2 categories of respondents to have used anxiolytics (*P* < .001) and antidepressants (*P* < .001) in the 12 months before data collection. They also estimate that their use of over-the-counter medications has increased over the same period (*P* < .001).

### Relative Risk of Health Status Deterioration Based on the Level of Exposure

As shown in Table 3, the participants’ health status varied based on the degree of exposure to the disaster. The results show significant disparities between respondents highly or moderately exposed to the train derailment and those not exposed. The exposed participants were twice as likely to consider their state of health to be poor (OR = 2.16, CI: 1.24-3.76) and to believe that their state of health had deteriorated in the last 3 years (OR = 2.40, CI; 1.54-3.75). The data also indicate that victims of the train derailment were 3 times more likely to have experienced, during the 12 months preceding the survey, a period of depression (OR = 3.00, CI: 1.92-4.69) and 4 times more likely to have a high level of psychological distress (OR = 3.99) and a mood disorder (OR = 4.75, CI: 1.89-11.94). A significantly higher proportion of the victims of the train derailment also presented manifestations of post-traumatic stress (OR = 10.13, CI: 5.83-17.59), an anxiety disorder (OR = 7.40, CI: 2.97-18.43) or post-traumatic growth (OR = 4.64, CI: 2.93-7.34), compared to the non-disaster victims.

**Table 3.** Odds Ratio (OR) of Dichotomous Health Variables by Exposure to Train Derailment (%) (N = 800).

Variables	Exposure level			Odds Ratio (OR) (CI 95%)
	Exposed (n = 624)	Unexposed (n = 176)	Significance level (Khi-square) χ2	
Perceived health status			7.78***	2.16 (1.24-3.76)
Fair to poor	17.8	9.1		
Excellent to very good	82.2	90.9		
Changes in health level			15.65***	2.4 (1.54-3.75)
Has deteriorated	30.3	15.3		
Has improved/Remained stable	69.7	84.7		
State of post-traumatic stress^a^			91.87***	10.13 (5.83-17.59)
Yes (26 and over)	48.6	8.5		
No (25 and less)	51.4	91.5		
Mood disorders			13.19***	4.75 (1.89-11.94)
Yes	12.2	2.8		
No	87.8	97.2		
Anxiety disorder			24.74***	7.4 (2.97-18.43)
Yes	17.8	2.8		
No	82.2	97.2		
High psychological distress^b^			31.06***	3.99 (2.38-6.69)
Yes (7 and over)	31.3	10.2		
No (6 and less)	68.8	89.8		
Depressive episode			24.68***	3 (1.92-4.69)
Yes	34.2	14.8		
No	65.8	85.2		
Post-traumatic growth^c^			48.98***	4.64 (2.93-7.34)
Yes (57 and over)	42.3	13.6		
No (56 and less)	57.7	86.4		

*Note*. CI = confidence interval.

a Horowitz’s Impact of Event Scale.

b Kessler Psychological Distress Scale.

c (PTGI) Post-Traumatic Growth Inventory.

*** *P* < .001.

The odds ratio analyses for the alcohol and drug consumption variables (Table 3) show that the victims of the train derailment were twice as likely as the non-exposed victims (1) to have increased their consumption of alcohol since this disaster (OR = 2.66, CI: 1.19-5.93) and (2) to have taken anxiolytics in the past 12 months (OR = 2.22, CI: 1.32-3.71). Train derailment victims were also 3 times more likely than non-disaster victims to have used antidepressants (OR = 2.92, CI: 1.48-5.74) in the same period (past 12 months) and they were 6 times more likely to have increased their consumption of non-prescription drugs since the tragedy (OR = 6.47, CI: 2.01-20.88).

### Predictive Factors of Post-Traumatic Stress Disorder

According to Nagelkerke’s *R*^2^, the complete model explains 37.3% of the symptoms of post-traumatic stress (Table 4). While the first 2 blocks have a share of only 0.2% and 5.1%, respectively, the exposure-related variables explain 32.0% of the onset of post-traumatic stress. Among the socio-demographic characteristic variables, only age is significant (*B* = 0.824, *P* < .001). According to the rating reports (*Exp [B]* in the tables), people aged 65 and over are twice as likely to experience symptoms of post-traumatic stress disorder (PTSD). Dissatisfaction with the assistance received is also linked to symptoms of post-traumatic stress (*B* = 0.465, *P* = .036). The concern experienced by respondents during the exposure or, more specifically, fear for their own life or that of a loved one (*B* = 0.805, *P* < .001) and the absence of news from a loved one for a few hours or a few days following the train derailment (*B* = 0.515 *P* = .007) are significant explanatory factors for post-traumatic stress. In fact, having feared for one’s own life or that of a loved one doubles the risk of developing PTSD (*B* = 0.805 *P* < .001). A respondent who had injuries or who had a loved one who was injured during the disaster was twice as likely to experience post-traumatic stress disorder (*B* = 0.712, *P* = .022). In addition to these variables, having been relocated (*B* = 0.448, *P* = .019) and considering the train derailment to have been a stressful event with negative impacts on their lives (negative perception of the tragedy) (*B* = 1.753, *P* < .001) are significant variables in the model. Maintaining a negative perception of the event therefore increases the risk of post-traumatic stress disorder more than fivefold.

**Table 4.** Hierarchical Logistic Regression Analysis of Post-Traumatic Stress and Psychological Distress as a Function of Perceived Social Support and Exposure Characteristics (N = 800).

Variables	Post-traumatic stress				Psychological distress			
	B	*P*	Exp(B) (CI 95%)	*R* ^2^	B	*P*	Exp(B) (CI 95%)	*R* ^2^
Constant	–2.917	0	0.054		–1.679	0	0.187	
Block 1. Socio-demographic characteristics				.002				.04
Age (1 = 65 year old and over)	0.824	.000***	2.281 (1.514-3.434)		–0.315	.143	0.73 (0.478-1.113)	
Marital status (1 = Married)	–0.094	.615	0.91 (0.631-1.314))		–0.68	.000***	0.507 (0.351-0.731)	
Block 2. Social support								
Social support (1 = poor)	0.055	.885	1.057 (0.500-2.236)	.051	1.907	.000***	6.73 (3.100-14.612)	.093
Satisfaction with assistance received (1 = Dissatisfied)	0.465	.036*	1.592 (1.031-2.458)		0.56	.009***	1.751 (1.149-2.668)	
Block 3. Variables on exposure								
Fear for one’s own life or that of a loved one (1 = Yes)	0.805	.000***	2.238 (1.482-3.380)	.32	0.414	.065	1.513 (0.974-2.349)	.09
No news from a loved one (1 = Yes)	0.515	.007***	1.674 (1.151-2.437)		0.299	.135	1.349 (0.911-1.998)	
Injured or a loved one was injured (1 = Yes)	0.712	.022*	2.039 (1.108-3.751)		1.171	.000***	3.224 (1.821-5.709)	
Death of a loved one (1 = Yes)	0.208	.283	1.231 (0.842-1.799)		–0.035	.865	0.966 (0.650-1.437)	
Job loss (1 = Yes)	0.26	.32	1.297 (0.777-2.165)		–0.136	.613	0.873 (0.515-1.480)	
Relocation (1 = Yes)	0.448	.019*	1.566 (1.078-2.275)		0.253	.205	1.287 (0.871-1.902)	
Negative perception of the derailment (1 = Yes)	1.753	.000***	5.771 (3.750-8.881)		0.553	.013*	1.739 (1.122-2.695)	
Total model	Khi^2^ = 252.042		*P* < .001	*R*^2^ = 0.373***	Khi^2^ = 129.832		*P* < .001	*R*^2^ = .223***

*Note*. B = Unstandardized Beta; *p = *Probability value; Exp(B) = Odds ratio; CI = Confidence interval; *R*^2^ = Nagelkerke squared correlation coefficient.

* *P* < .05. ***P < .001.

### Predictive Factors of Psychological Distress

With regard to psychological distress, the logistic regression model explains 22.3% of the variance (Table 4). Marital status is negatively correlated with the dependent variable, and psychological distress is less common among respondents who are legally married or living together (*B* = −0.680, *P* < .001). In addition, psychological distress is strongly correlated with low perceived social support (*B* = 1.907, *P* < .001). According to rating reports, low perceived social support increases the risk of experiencing psychological distress by more than 6 times. Furthermore, experiencing ongoing dissatisfaction with the help received following the disaster involves almost twice the risk of psychological distress (*B* = 0.560, *P* = .009). The exposure-related variables explain 9.0% of psychological distress, which is relatively similar to the variables related to social support (9.3%). A person who was injured or who had a loved one who suffered injuries (*B* = 1.171, *P* < .001) was 3 times more likely to experience psychological distress. Finally, a respondent with a negative perception of the event was more likely to experience psychological distress (*B* = 0.553, *P* = .013).

### Predictive Factors of Depressive Episode

According to the regression model, 19.6% of the variance of depressive episodes can be explained by the variables studied: 2.3% by socio-demographic characteristics, 10.8% by social support and 6.5% by exposure to the disaster (Table 5). Being married is negatively correlated with having a depressive episode (*B* = −0.591, *P* = .001), which suggests that the relationship is a protective factor. In addition, low social support leads to more than 5 times the risk of experiencing a depressive episode (*B* = 1.754, *P* < .001). Furthermore, having been dissatisfied with the help received doubles the risk of experiencing a depressive episode (*B* = 0.894, *P* < .001). Among the exposure-related variables, only the negative perception of the derailment is correlated with a depressive episode, more than doubling the risk of experiencing this problem (*B* = 0.904, *P* < .001).

**Table 5.** Hierarchical Logistic Regressions Analysis on Depressive Episode and Post-Traumatic Growth as a Function of Perceived Social Support and Exposure Characteristics(N = 800).

Variables	Depressive episode				Post-traumatic growth			
	B	*P*	Exp(B) (CI 95%)	*R* ^2^	B	*P*	Exp(B) (CI 95%)	*R* ^2^
Constant	–1.695	0	0.184		–1.69	0		
Block 1. Socio-demographic characteristics				.023				.002
Age (1 = 65 year old and over)	0.178	.369	1.194 (0.810-1.760)		.28	.128	1.323 (0.923-1.897)	
Marital status (1 = Married)	–0.591	.001***	0.554 (0.391-0.784)		.035	.833	1.036 (0.745-1.441)	
Block 2. Social support								
Social support (1 = poor)	1.754	.000***	5.775 (2.647-12.599)	.108	–0.986	.016*	0.373 (0.167-0.834)	.019
Satisfaction with assistance received (1 = Dissatisfied)	0.894	.000***	2.444 (1.631-3.663)		0.144	.482	1.155 (0.773-1.727)	
Block 3. Variables on the exposure								
Fear for one’s own life or that of a loved one (1 = Yes)	0.371	.078	1.45 (0.959-2.192)	.065	0.065	.739	1.067 (0.730-1.559)	.117
No news from a loved one (1 = Yes)	–0.085	.664	0.919 (0.627-1.347)		0.258	.153	1.294 (0.909-1.843)	
Injured or a loved one was injured (1 = Yes)	0.309	.286	1.362 (0.772-2.401)		0.219	.429	1.245 (0.723-2.144)	
Death of a loved one (1 = Yes)	0.147	.443	1.159 (0.795-1.689)		0.409	.022*	1.505 (1.062-2.132)	
Job loss (1 = Yes)	–0.072	.783	0.93 (0.557-1.554)		0.177	.465	1.193 (0.742-1.918)	
Relocation (1 = Yes)	–0.069	.722	0.934 (0.639-1.363)		0.43	.016*	1.537 (1.084-2.178)	
Negative perception of the derailment (1 = Yes)	0.904	.000***	2.47 (1.625-3.753)		0.872	.000***	2.392 (1.638-3.492)	
Total model	Khi^2^ = 116.232		*P* < .001	*R*^2^ = .196***	Khi^2^ = 82.643		*P* < .001	*R*^2^ = .138***

*Note.* B = unstandardized beta; *p = *probability value; Exp(B) = odds ratio; CI = confidence interval; *R*^2^ = Nagelkerke squared correlation coefficient.

* *P* < .05. ***P < .001.

### Predictive Factors of Post-Traumatic Growth

The variables examined explain only 13.8% of the presence of post-traumatic growth. Socio-demographic characteristics and social support explain, respectively, 0.2% and 1.9% of the variance of post-traumatic growth, whereas the variables linked to exposure explain 11.7% (Table 5). The socio-demographic characteristics present in the model are not linked to post-traumatic growth, whereas low social support is negatively linked to this variable (*B* = −0.986, *P* < .001). On the other hand, the death of a loved one (*B* = 0.409, *P* = .022) and having been relocated (*B* = 0.430, *P* = .016) are correlated with post-traumatic growth. Finally, a person with a negative perception of the event is twice as likely to experience post-traumatic growth, according to the PTGI (*B* = 0.872, *P* < .001).

### Predictive Factors of Increased Alcohol Consumption

According to Nagelkerke R^2^, socio-demographic variables, social support and exposure to the disaster explain 17.8% of the increase in alcohol consumption (Table 6). More specifically, being over 65 years of age is negatively correlated with higher consumption following the tragedy (*B* = −1.395, *P* < .01). This suggests that it is people under the age of 65 who are struggling with this habit. Of the exposure variables, only the presence of a negative perception of the event raises the risk of increased alcohol consumption by almost 5 times. (*B* = 1.555, *P* < .01).

**Table 6.** Hierarchical Logistic Regressions Analysis on Respondents’ Consumption of Alcohol and Non-Prescription Drugs.

Variables	Alcohol consumption				Consumption of non-prescription drugs			
	B	*P*	Exp(B) (CI 95%)	*R* ^2^	B	*P*	Exp(B) (CI 95%)	*R* ^2^
Constant	–3.711	0	0.024		–4.795	0	0.008	
Block 1. Socio-demographic characteristics				.061				.005
Age (1 = 65 year old and more)	–1.395	.004**	0.248 (0.095-0.646)		.219	0.525	1.245 (0.634-2.444)	
Marital status (1 = Married)	–0.319	.261	0.727 (0.416-1.269)		.244	0.426	1.276 (0.700-2.329)	
Block 2. Social support								
Social support (1 = Poor)	0.613	.185	1.846 (0.745-4.576)	.026	1.767	.000***	5.854 (2.573-13.315)	.076
Satisfaction with assistance received (1 = Dissatisfied)	0.36	.24	1.434 (0.786-2.614)		0.368	.234	1.444 (0.789-2.644)	
Block 3. Variables based on the level of exposure								
Fear for one’s own life or that of a loved one (1 = Yes)	0.695	.071	2.003 (0.942-4.260)	.003	0.88	.030*	2.411 (1.091-5.328)	.093
No news from a loved one (1 = Yes)	–0.138	.635	0.871 (0.493-1.540)		0.266	.365	1.305 (0.733-2.323	
Injured or a loved one was injured (1 = Yes)	0.45	.236	1.568 (0.745-3.301)		–0.032	.941	0.968 (0.411-2.281)	
Death of a loved one (1 = Yes)	0.077	.794	1.08 (0.606-1.924)		–0.091	.763	0913 (0.506-1.648)	
Job loss (1 = Yes)	0.113	.742	1.12 (0.571-2.197)		0.238	.526	1.269 (0.608-2.649)	
Relocation (1 = Yes)	–0.315	.289	0.73 (0.408-1.307)		0.064	.826	1.066 (0.604-1.880)	
Negative perception of the derailment (1 = Yes)	1.555	.001**	4.735 (1901-11.794)		1.471	.003**	4.353 (1.671-11.340)	
Total model	Khi^2^ = 64.216		P < .001	*R*^2^ = .178***	Khi^2^ = 63.211		*P* < .001	*R*^2^ = .179

*Note.* B = unstandardized Beta; *p = *probability value; Exp(B) = odds ratio; CI = confidence interval; *R*^2^ = Nagelkerke squared correlation coefficient.

* *P* < .05. **P < .01. ***P < .001.

### Predictive Factors of Increased Use of Non-Prescription Drugs

The variables included in our regression analysis explain 17.9% of the presence of greater use of non-prescription drugs (Table 6). No socio-demographic variables were retained as significant, however. Moreover, people with low social support presented a six-fold increase in the risk of using more drugs (*B* = 1.767, *P* < .001). Similar to its effect on alcohol consumption, a negative perception of the derailment increased the likelihood of drug use by nearly 4 times (*B* = 1.471, *P* < .01). Finally, fearing for one’s life or the life of a loved one doubled the risk of causing a change in the use of non-prescription drugs (*B* = 0.800, *P* < .001).

## Discussion

This research shows that, 3 years after a train derailment, highly exposed people were still experiencing more difficulties than moderately or non-exposed people. This study, like others, reveals that the individuals who were highly exposed to the train derailment have a higher incidence of post-traumatic stress disorder,^3,4^ psychological distress^7^ and mood or anxiety disorders.^6^ It is also not surprising to find that people who were highly exposed to the train derailment are significantly more likely than moderately or non-exposed people to have consumed anxiolytics and antidepressants in the 12 months prior to the data collection. Highly exposed individuals also estimate that their consumption of prescription drugs increased over the same period. Other studies have also revealed that psychological symptoms, following a disaster, can lead to an increase in the number of psychotropic prescriptions.^33-35^ However, unlike other studies conducted on the subject,^27-29^ this study does not point to a clear correlation between the level of exposure to the disaster and weekly alcohol abuse among the respondents. Nevertheless, those who are highly exposed are more likely than others to estimate that their alcohol consumption has increased over the past 3 years. It is therefore possible to postulate, as other authors have done,^31,32^ that alcohol can be used as self-medication to relieve the pain or weight of painful memories in some victims of the Lac-Mégantic tragedy. The relationship between the level of disaster exposure and the psychological health variables remains significant, even when the other possible correlates, that is, socio-demographic and social support variables, are included in the same regression model. This supports the idea that exposure to a technological disaster is likely to have a significant impact on those affected, even when they have protective factors such as good social support to cope with daily stress or satisfaction with the assistance received during the disaster. The regression models do indicate a low level of explanation for post-traumatic growth and depressive episodes, however. This could indicate that these psychological dimensions are more sensitive to other events not measured in the context of this study. Regarding post-traumatic growth, the time gap between the disaster and the data collection may have been insufficient for individuals to be able to see positive aspects related to trauma in their daily lives.

Although it would have been very useful to know the respondents’ health status before the derailment occurred, the data collected seem to point in the same direction for most of the variables studied. People who suffered both human and property losses and who feel that the train derailment caused them to lose something important in their lives have a more negative perception of their physical health and have multiple consequences in terms of psychological symptoms, including post-traumatic stress disorder, mood disorders, anxiety disorders or psychological distress. Considering this information, it is surprising that so few people highly or moderately exposed to the train tragedy consulted a psychologist or social worker in the 12 months preceding this survey.

## Study Strengths and Limitations

This study has several strengths, notably the use of a random sampling method. The high response rate and the use of previously validated questions are also strengths of this study. Finally, the high number of respondents in the 3 groups of participants is a positive factor in the internal validity of the results. Although the results support the importance of integrating diverse variables to portray the impact of disasters on the overall health of adults, the results cannot be generalized to all those exposed to the train derailment or other types of disasters. It is also possible that people who refused to participate to this study had precarious socio-demographic characteristics and health status compared to those who volunteered. It is also possible that those exposed to the train derailment who agreed to participate in this study were better able to cope with the different stresses experienced than those who refused to complete the telephone questionnaire. The high number of respondents is a positive factor in the internal validity of the results, however.

Some respondents may not have been completely honest about their mental health status, especially the questions about mood, anxiety or depression issues. In addition, the lack of pre-disaster data with respect to the respondents’ health status and the fact that the data collection was performed more than 3 years after the train derailment are limitations that do not allow us to conclude that exposure to this disaster is the only traumatic event causing physical and psychosocial problems for the victims of the train derailment. People may have experienced other personal, marital, family, professional or social events that forced them to question their values, beliefs and lifestyle and that led to our findings. In order to avoid this limitation, future studies of long-term impacts after a disaster should control for multiple traumatic experiences.

## Conclusion

The main findings of this study can probably be explained, at least in part, by the fact that some of the victims perceived the train tragedy as a catastrophe originating in the negligence of a rail company with little concern for safety. Poor rail maintenance, as well as the lack of rail safety regulations by government authorities, can also explain our results. It is also possible that, 3 years after the train derailment, the presence of various significant differences between the victims and the non-exposed individuals is because many corrective measures have still not been taken within the community, despite all the steps and efforts made by the municipal and health authorities. Studies of the consequences of other train derailments and industrial accidents that caused major damage to community infrastructure and personal property have come to the same conclusions either a few months or years after such events.^4,10,11^ It is therefore paramount, in this context, to ensure that victims of technological disasters have access to health and social services based on the main tenets of post-disaster crisis intervention, including meeting the needs of victims by being present in the main places frequented by them.^48-50^ This is even more relevant given that the more time that elapses before care is provided to victims following a disaster, the more likely it is that their physical and mental condition will deteriorate.^51,52^

## Notes

i. The municipality of Lac-Mégantic is located in Québec (Canada). In this municipality, 40.8% of the population lives in rental housing. The proportion of households with children is 28.7%, while that of single-parent families with children is 34.6%. Nearly a third of the men (32.5%) and a quarter of the women (25.7%) aged 25 to 64 have a level of education lower than a high-school diploma. More than half (54.6%) of residents aged 15 and over are gainfully employed, while 19.8% of 18 to 64 years-old live below the low-income threshold. This percentage is 21.1% for people aged 65 and over.
